# Frozen/thawed meat quality associated with muscle fiber characteristics of porcine *longissimus thoracis et lumborum*, *psoas major*, *semimembranosus,* and *semitendinosus muscles*

**DOI:** 10.1038/s41598-021-92908-3

**Published:** 2021-06-25

**Authors:** Huilin Cheng, Sumin Song, Gap-Don Kim

**Affiliations:** 1 Graduate School of International Agricultural Technology, Seoul National University, Pyeongchang, 25354 Republic of Korea; 2 Institutes of Green Bio Science and Technology, Seoul National University, Pyeongchang, 25354 Republic of Korea

**Keywords:** Biological techniques, Biotechnology, Cell biology

## Abstract

To evaluate the relationship between muscle fiber characteristics and the quality of frozen/thawed pork meat, four different muscles, M. *longissimus thoracis et lumborum* (LTL), M. *psoas major* (PM), M. *semimembranosus* (SM), and M. *semitendinosus* (ST), were analyzed from twenty carcasses. Meat color values (lightness, redness, yellowness, chroma, and hue) changed due to freezing/thawing in LTL, which showed larger IIAX, IIX, and IIXB fibers than found in SM (*P* < 0.05). SM and ST showed a significant decrease in purge loss and an increase in shear force caused by freezing/thawing (*P* < 0.05). Compared with LTL, SM contains more type IIXB muscle fibers and ST had larger muscle fibers I and IIA (*P* < 0.05). PM was the most stable of all muscles, since only its yellowness and chroma were affected by freezing/thawing (*P* < 0.05). These results suggest that pork muscle fiber characteristics of individual cuts must be considered to avoid quality deterioration during frozen storage.

## Introduction

Postmortem metabolic changes affect meat quality characteristics such as pH, color, water-holding capacity, tenderness, and texture properties. Muscle fiber characteristics (fiber type distribution, size, density, and relative composition) are considered critical meat quality factors regardless of animal species, breed, gender, and growth performance^1–6^. In addition, individual skeletal muscles have unique structural and functional properties that are also determined by different muscle fiber types^7,8^. After conversion to meat, muscle fiber characteristics influence the quality among the meat cuts^9–12^. Studies have widely demonstrated the relationship between muscle fiber characteristics and meat quality. However, such studies are limited to fresh meat. In contrast, few studies have addressed the relationship between aged or frozen/thawed meat quality and muscle fiber characteristics^13,14^.

Freezing is frequently used to ensure the safety and quality of meat during storage. Nevertheless, freezing can affect meat quality by causing distortion of tissue structure, protein denaturation, lipid oxidation, and exudates during thawing^15–17^. To minimize quality deterioration owing to freezing/thawing, improved technologies have been proposed^18–20^. However, physical and biochemical changes during freezing or thawing are inevitable. We have previously analyzed the effect of freezing/thawing on the microstructure of muscle^14^, and we found differences in freezing-susceptibility among muscle fiber types. In addition, muscle fiber type distribution varies among different muscles^9,10,12^. Therefore, it is expected that the quality of each type of meat will be affected differently by freezing/thawing. However, the relationship between muscle fiber characteristics and frozen/thawed meat quality is unknown.

In this study, four types of porcine skeletal muscles, M. *longissimus thoracis et lumborum* (loin), M. *psoas major* (tenderloin), M. *semimembranosus* (inside of ham), and M. *semitendinosus* (eye of ham), were studied to evaluate the effect of freezing/thawing on their quality characteristics; and determine the relationship between quality changes and muscle fiber characteristics.

## Results

### Comparison of proximate composition among different pork cuts

Table 1 shows the proximate composition of different pork cuts, including moisture, IMF, crude protein, and crude ash contents. Several pork cuts showed significant differences in moisture and IMF content (*P* < 0.05). However, there was no significant difference in crude protein and ash content among all cuts (*P* > 0.05). Moisture content was lowest in LTL and IMF content was higher in LTL and SM than in PM and ST (*P* < 0.05). PM had the highest moisture content and the lowest IMF content (*P* < 0.05). There was no significant difference in moisture and IMF content between SM and ST (*P* > 0.05).

**Table 1:** Proximate composition of porcine skeletal muscles.

Measurements	Muscles^1)^			
	LTL	PM	SM	ST
Moisture (%)	71.82 ± 0.25^c^	74.76 ± 0.39^a^	73.25 ± 0.62^b^	73.83 ± 0.08^b^
Intramuscular fat (%)	3.96 ± 0.39^a^	1.91 ± 0.29^c^	3.14 ± 0.76^ab^	2.78 ± 0.17^b^
Crude protein (%)	22.91 ± 0.35	22.28 ± 0.16	21.16 ± 0.82	22.14 ± 0.37
Crude ash (%)	1.12 ± 0.15	1.07 ± 0.18	1.17 ± 0.35	1.20 ± 0.19

^a–c^Means ± SE with different superscript within the same row are significantly different at *P* < 0.05.

^1)^LTL, M. *longissimus thoracis et lumborum*; PM, M. *psoas major*; SM, M. *semimembranosus*; ST, M. *semitendinosus.*

### Comparison of meat quality changes caused by freezing/thawing among different pork cuts

The pH of pork ranged from 5.51 to 5.81; however, no significant effects of cut and freezing/thawing on pH were observed (*P* > 0.05; Table 2). Meat color was compared between fresh and frozen/thawed pork cuts by using lightness (CIE L*), redness (CIE a*), yellowness (CIE b*), chroma, and hue values. Our results showed significant color differences between fresh and frozen/thawed meat cuts (*P* < 0.01; Table 2). Fresh LTL and SM had higher CIE L* values compared to those from fresh PM and ST (*P* < 0.0001). PM had the lowest CIE L* among all fresh meat cuts (*P* < 0.0001). Freezing/thawing decreased CIE L* in LTL and ST (*P* < 0.05), but had no effect on PM and SM (*P* > 0.05). Among all fresh pork cuts, PM showed the highest CIE a*, whereas LTL had the lowest CIE a* (*P* < 0.0001). In addition, freezing/thawing increased CIE a* in LTL and SM (*P* < 0.05), and had no effect on PM and ST (*P* > 0.05). CIE a* was lower in fresh SM than in fresh ST (*P* < 0.05), but there was no difference between frozen/thawed SM and ST (*P* > 0.05). Fresh and frozen/thawed SM showed the highest CIE b* among all samples (*P* < 0.01). Except for SM (*P* > 0.05), CIE b* was affected by freezing/thawing in all other pork cuts, as CIE b* increased in LTL and decreased in PM and ST (*P* < 0.05). In contrast, fresh and frozen/thawed LTL and PM showed a contrary trend in chroma and hue values than that observed for CIE b*, i.e., LTL had the lowest chroma and the highest hue values in fresh and frozen/thawed groups, respectively, whereas PM showed the highest chroma and the lowest hue values in each group (*P* < 0.0001). Both the chroma and hue values were higher in SM than in ST regardless of freezing/thawing (*P* < 0.05). Freezing/thawing did not affect the chroma values in SM and ST (*P* > 0.05). However, the chroma values in LTL (*P* < 0.0001) and PM (*P* < 0.05) were affected by freezing/thawing, indicating an increase in LTL but a decrease in PM. The hue values decreased in LTL, SM, and ST after freezing/thawing (*P* < 0.05), but the hue value of PM was not affected by freezing/thawing (*P* > 0.05). All color traits showed the combined effect of freezing/thawing and muscle type (*P* < 0.05). Based on the muscle type, each color trait showed a different trend after freezing/thawing as follows: CIE L* did not change in SM; CIE a* did not change in PM and ST; CIE b* increased in LTL (contrary to the other muscles); chroma value did not change in ST and SM; and hue value did not change in PM.

**Table 2:** Comparison of pH, meat color, water-holding capacity, and shear force between fresh and frozen/thawed pork in different muscles.

Measurements	Muscles^1)^									Levels of significance^2)^						
	LTL		PM		SM		ST		PSE^3)^	Muscle		FT				FT × Muscle
	Fresh	FT	Fresh	FT	Fresh	FT	Fresh	FT		Fresh	FT	LTL	PM	SM	ST	
pH	5.62	5.67	5.81	5.77	5.76	5.59	5.51	5.60	0.12	ns	ns	ns	ns	ns	ns	ns
Lightness (CIE L*)	62.51^a^	58.43^b^	44.73^d^	42.40^d^	62.56^a^	60.29^ab^	58.71^b^	53.05^c^	1.23	***	***	*	ns	ns	*	**
Redness (CIE a*)	4.42^e^	6.32^d^	18.66^a^	17.26^a^	8.86^c^	10.87^b^	9.54^b^	10.56^b^	0.52	***	***	***	ns	*	ns	*
Yellowness (CIE b*)	6.09^f^	6.95^e^	9.83^b^	7.79^d^	11.47^a^	10.23^ab^	8.82^c^	7.96^d^	0.40	***	**	**	*	ns	**	**
Chroma (C)	7.53^f^	9.41^e^	21.15^a^	18.95^b^	14.50^c^	14.93^c^	13.10^d^	13.24^d^	0.56	***	***	***	*	ns	ns	*
Hue (h)	54.12^a^	47.62^c^	27.26^f^	24.03^f^	52.38^b^	43.22^d^	43.40^d^	37.30^e^	1.36	***	***	**	ns	***	*	**
WBSF^4)^ (kg/cm^2^)	2.15^d^	2.17^d^	2.12^d^	2.23^d^	2.86^c^	3.24^b^	2.89^c^	3.74^a^	0.51	*	**	ns	ns	*	*	**
Purge loss (%)	2.49^e^	3.99^c^	2.14^e^	3.08^d^	3.85^c^	7.26^a^	5.23^b^	8.46^a^	0.62	*	*	*	ns	*	***	**
Drip loss (%)	0.94^bc^	0.83^c^	0.87^c^	0.77^c^	1.26^a^	0.79^c^	1.06^b^	0.82^c^	0.10	*	ns	ns	ns	***	*	ns
Cooking loss (%)	27.34^d^	29.51^c^	27.81^d^	28.53^ cd^	31.42^bc^	31.08^b^	34.06^a^	33.28^a^	1.01	*	*	*	ns	ns	ns	ns

^1)^LTL, M. *longissimus thoracis et lumborum*; PM, M. *psoas major*; SM, M. *semimembranosus*; ST, M. *semitendinosus.*

^2)^ns, not significant; *P < 0.05; **P < 0.01; ***P < 0.0001; FT, freezing/thawing; FT × Muscle, combination effect of freezing/thawing and muscle type.

^3)^Pooled SE.

^4)^Warner–Bratzler shear force.

^a–f^ Means with same superscripts are not significantly different (*P* < 0.05).

The interaction between freezing/thawing and muscle type was also observed in WBSF (*P* < 0.01) (Table 2). Fresh SM and ST showed higher WBSF values than LTL and PM (*P* < 0.05). In addition, freezing/thawing increased WBSF values (*P* < 0.05) of SM and ST; whereas, WBSF did not change in LTL and PM after freezing/thawing (*P* > 0.05). Due to freezing/thawing, WBSF was higher in SM or ST than in LTL and PM (*P* < 0.01). The WBSF values of ST and SM were changed but those of LTL and PM were not altered by freezing/thawing.

The results of purge loss, drip loss, and cooking loss were reported to assess the water-holding capacity of pork (Table 2). The combined effect of freezing/thawing and muscle type was only manifested in purge loss (*P* < 0.01). Based on the muscle type, purge loss was not changed or its values varied among the muscles after freezing/thawing. Specifically, fresh and frozen/thawed SM and ST showed higher purge loss values than those observed in LTL and PM, regardless of freezing–thawing (*P* < 0.05). Fresh ST had higher purge loss than SM (*P* < 0.05), whereas there was no difference between frozen/thawed SM and ST (*P* > 0.05). Freezing/thawing increased purge loss in LTL, SM, and ST (*P* < 0.05), whereas no significant effect on PM purge loss was found after freezing/thawing (*P* > 0.05). Drip loss demonstrated a significant difference between the fresh pork cuts (*P* < 0.05). The value was highest in fresh SM, while it was lowest in fresh PM. However, there were no significant differences between the frozen/thawed pork cuts (*P* > 0.05). Freezing/thawing affected drip loss in SM and ST (decreased) (*P* < 0.0001). ST showed the highest weight loss after cooking among all fresh pork cuts (*P* < 0.05). SM had higher cooking loss than LTL and PM (*P* < 0.05). The cooking loss due to freezing/thawing was higher in and ST than the cooking loss found in LTL, PM, and SM (*P* < 0.05). Among all pork cuts, only LTL showed a significant increase in cooking loss after freezing/thawing (*P* < 0.05).

### Comparison of MFCs among pork cuts

**Figure 1:**
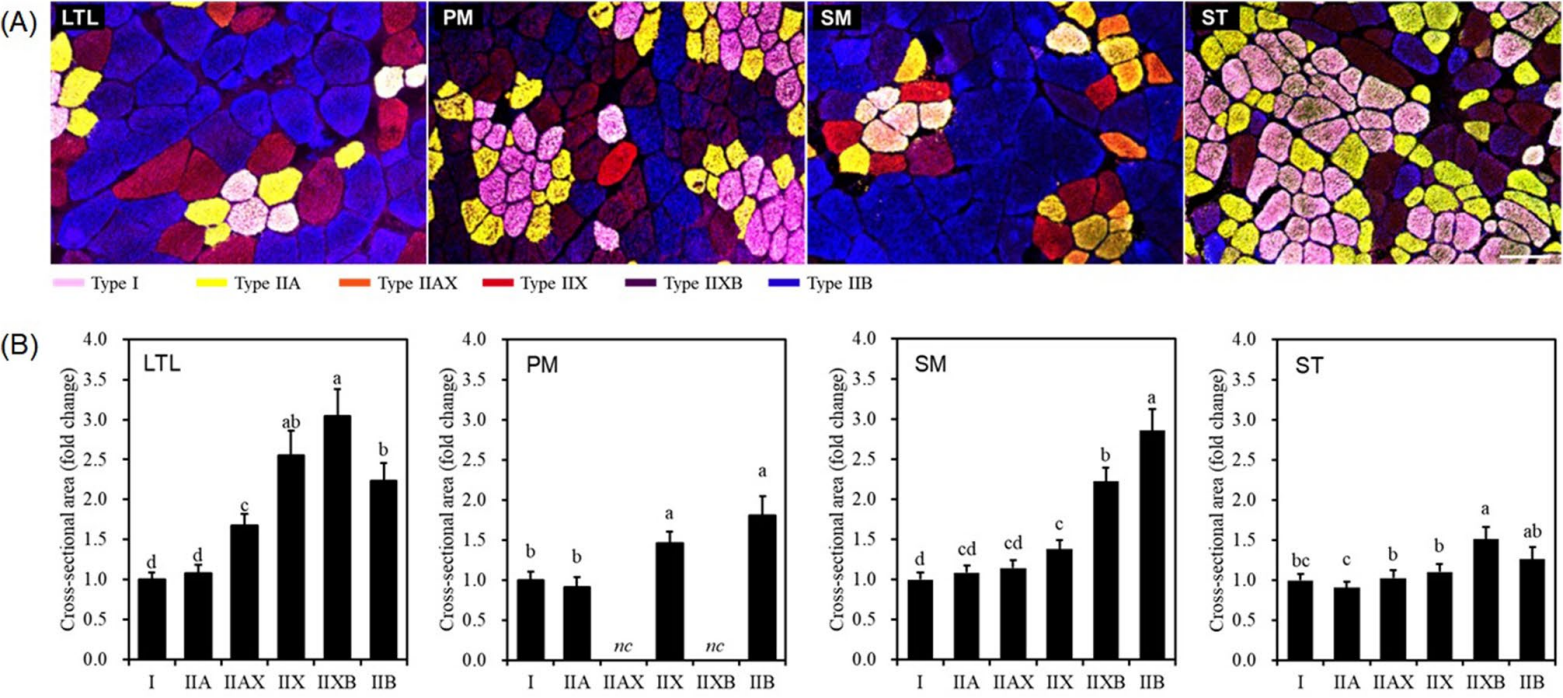
Representative stained cross-sections (**A**) and relative cross-sectional area (**B**) of pork skeletal muscles. LTL, M. *longissimus thoracis et lumborum*; PM, M. *psoas major*; SM, M. *semimembranosus*; ST, M. *semitendinosus*; *nc*, not considered. The merged images were obtained by incubation with BA-F8 (anti-myosin heavy chain (MHC) slow), SC-71 (anti-MHC 2a and 2x), BF-35 (anti-MHC slow and 2a), and BF-F3 (anti-MHC 2b). Bar = 100 µm. Different letters (a–d) on the bar in (**B**) indicate significant differences between muscle fiber types within the same muscle at *P* < 0.05.

**Figure 2:**
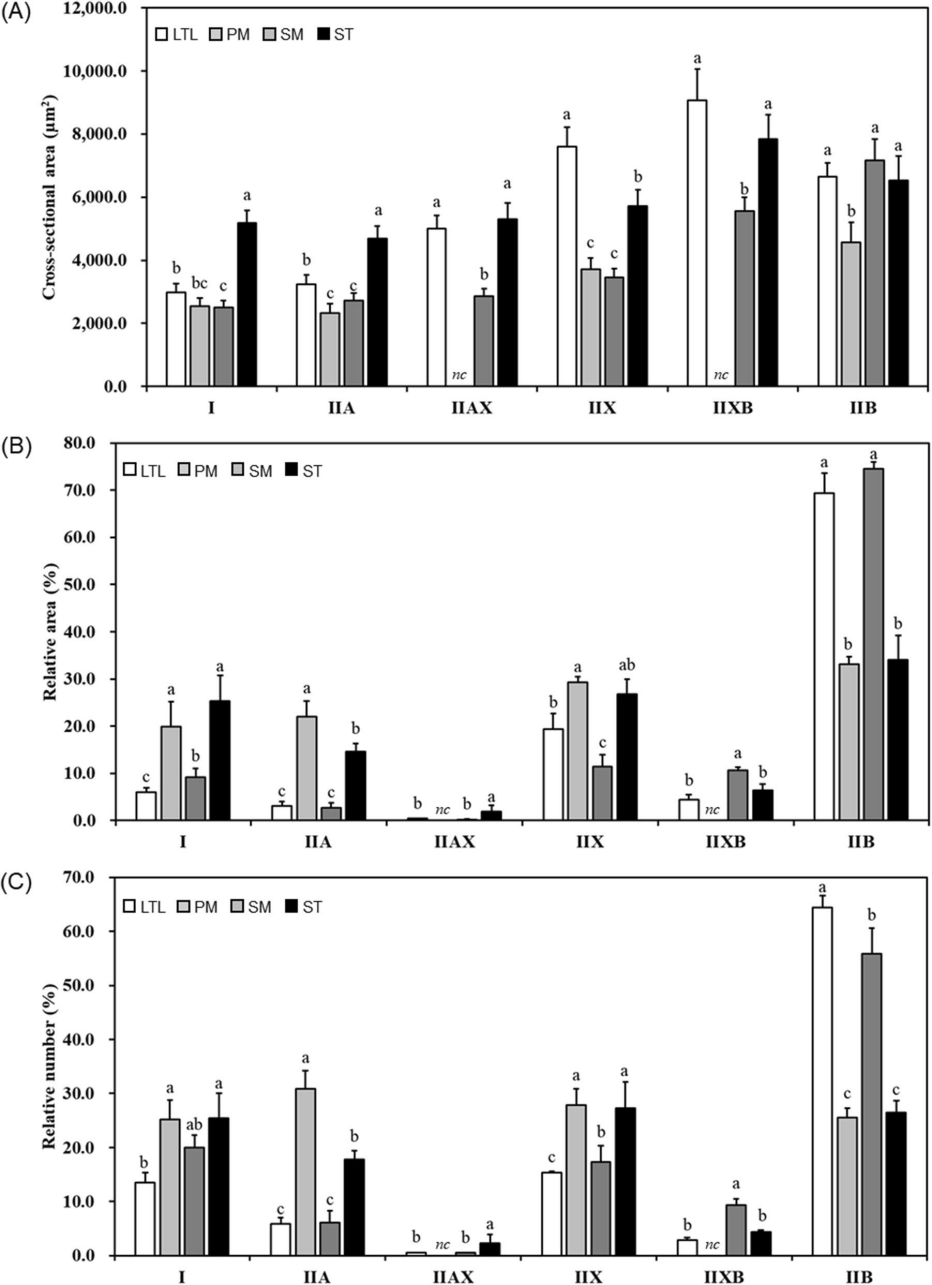
Comparison of muscle fiber characteristics between porcine skeletal muscles. (**A**) Cross-sectional area. (**B**) Relative area of muscle fiber. (**C**) Relative number of muscle fiber. LTL, M. *longissimus thoracis et lumborum*; PM, M. *psoas major*; SM, M. *semimembranosus*; ST, M. *semitendinosus*, *nc*, not considered. Muscle fiber types (I, IIA, IIAX, IIX, IIXB, and IIB) were classified by four anti-myosin heavy chain antibodies as shown in Fig. 1. Different letters (a–c) on the bar indicate significant differences between muscle types within the same muscle fiber type (*P* < 0.05).

The four pork cuts selected for this study consisted of a single muscle; specifically, LTL (loin), PM (tenderloin), SM (inside of ham), and ST (eye of ham). Six types of muscle fibers (I, IIA, IIAX, IIX, IIXB, and IIB) were detected based on the distribution of myosin heavy chains in muscle samples. Hybrid fiber types IIAX and IIXB were not considered in PM due to their low abundance (Fig. 1A). We determined the profiles of muscle fiber size for all four muscles, by measuring the CSA of each fiber type (Fig. 1B). We found that LTL, PM, and SM had larger IIX and IIB fibers than I and IIA fibers (*P* < 0.05), whereas no size difference was found between fiber types IIA and I (*P* > 0.05). In the SM, IIB muscle fibers were the largest, followed by IIXB muscle fibers, whereas in the LTL, the IIXB muscle fibers were larger than the IIB muscle fibers. In the ST, IIXB fibers were larger than I, IIA, IIAX, and IIX muscle fibers (*P* < 0.05); however, there was no size difference between IIXB and IIB fibers (*P* > 0.05). We found no size difference between fibers IIX and IIA in SM (*P* > 0.05). Conversely, all other muscles showed a significant size difference between fibers IIX and IIA (*P* < 0.05). Regarding the hybrid fibers IIAX and IIXB, IIXB was larger than IIAX in all muscles except for PM (*P* < 0.05). According to Fig. 2A, ST had larger I and IIA fibers than all other muscles (*P* < 0.05), and moreover, IIAX, IIXB, and IIB showed no size difference between ST and LTL (*P* > 0.05). PM had smaller I, IIA, IIX, and IIB fibers compared to those in ST (*P* < 0.05), whereas there were no significant size differences in fiber I, IIA, and IIX between PM and SM (*P* > 0.05).

Results of relative area (%) and relative number (%) showed a similar trend among all muscles (Fig. 2B,C). LTL had low values of fibers I, IIA, and IIX (*P* < 0.05) and a higher composition of IIB than that found in PM and SM (*P* < 0.05). SM showed no significant difference in type I composition than that observed in PM and ST (*P* > 0.05), but had lower compositions of types IIA and IIX (*P* < 0.05). In addition, SM showed a higher composition of fiber IIB, compared to that of PM or ST (*P* < 0.05). Relative area and number compositions of IIA were the highest in PM (*P* < 0.05), whereas relative area of type IIX was lowest in SM (*P* < 0.05). Regarding hybrid fiber types, ST had higher composition of type IIAX, whereas the composition of type IIXB was the highest in SM among all muscles (*P* < 0.05). No significant difference was found in composition of IIAX between LTL and SM (*P* > 0.05). However, relative area and relative number of IIXB were higher in SM compared to those found in LTL and in ST (*P* < 0.05).

### Relationship of MFC with the differences in meat quality between fresh and frozen-thawed pork

The pH, CIE L*, and cooking loss did not show any correlation with any trait of MFC (*P* > 0.05), whereas CIE a* and b*, chroma, hue, purge loss, drip loss, and WBSF were significantly correlated with MFC (*P* < 0.05) (Table 3). Specifically, CIE a* was negatively correlated with all MFC of type IIB (*P* < 0.05), but positively correlated with the relative number and relative area of type IIA (*P* < 0.01) and IIX (*P* < 0.05). CIE b* showed a similar trend in correlations with MFC to that in CIE a*, except for muscle fiber composition of type IIX. In addition, CIE b* was negatively correlated with the CSA of IIX and IIXB fibers (*P* < 0.05). The relationship between chroma and MFC was the same as that in CIE b*, except for the CSA of IIB fibers. Hue was positively correlated with the relative number of IIAX fibers and the relative area of types IIAX and IIX (*P* < 0.05). Purge loss was negatively correlated with MFC of type I and IIA (*P* < 0.05) and type IIX muscle fiber composition, whereas type IIB muscle fiber composition was positively correlated with purge loss (*P* < 0.01). Drip loss was negatively correlated with the CSA of type IIAX and IIXB and the relative area of type IIX but positively correlated with the CSA of type IIB and muscle fiber composition of type IIXB (*P* < 0.05). WBSF showed a relationship only with type IIAX: a positive correlation for CSA (*P* < 0.05) and a negative correlation for muscle fiber composition (*P* < 0.05).

**Table 3:** Correlation coefficients between muscle fiber characteristics and the difference in meat quality between fresh and frozen-thawed pork.

Muscle fiber characteristics		Meat quality traits									
Traits	Type	pH	Lightness (CIE L*)	Redness (CIE a*)	Yellowness (CIE b*)	Chroma	Hue	Purge loss	Drip loss	Cooking loss	WBSF^1)^
Cross − sectional area	I	− 0.36	0.16	− 0.10	− 0.09	− 0.13	0.01	− 0.61**	0.06	0.35	0.12
	IIA	− 0.42	0.25	− 0.34	− 0.21	− 0.30	− 0.18	− 0.46*	0.15	0.33	0.15
	IIAX	− 0.36	0.09	0.17	− 0.42	− 0.14	0.35	− 0.39	− 0.68**	− 0.08	0.46*
	IIX	− 0.39	0.05	− 0.36	− 0.66**	− 0.53*	0.06	0.11	− 0.33	− 0.22	0.29
	IIXB	− 0.41	− 0.04	0.09	− 0.52*	− 0.45*	0.37	− 0.14	− 0.84**	− 0.35	0.44
	IIB	− 0.06	− 0.03	− 0.52*	0.45*	− 0.38	− 0.38	0.29	0.51*	0.33	− 0.07
Relative number	I	− 0.13	0.11	0.37	0.41	0.35	0.03	− 0.58*	0.14	0.37	0.27
	IIA	− 0.04	− 0.10	0.75**	0.51*	0.68**	0.40	− 0.45*	− 0.30	0.14	0.23
	IIAX	− 0.11	− 0.14	0.30	− 0.02	0.11	0.45*	− 0.31	0.17	0.31	− 0.56*
	IIX	− 0.09	0.03	0.50*	0.35	0.43	0.40	− 0.60**	− 0.09	0.16	− 0.06
	IIXB	0.41	− 0.05	− 0.19	0.43	0.14	− 0.35	0.19	0.74**	0.27	− 0.42
	IIB	0.08	0.00	− 0.60**	− 0.47*	− 0.55*	− 0.36	0.70**	0.23	− 0.16	− 0.11
Relative area	I	− 0.36	0.23	0.34	0.43	0.44	0.06	− 0.74**	0.01	0.35	0.34
	IIA	− 0.12	− 0.04	0.71**	0.50*	0.66**	0.39	− 0.53*	− 0.29	0.16	0.24
	IIAX	− 0.20	− 0.11	0.29	− 0.08	0.07	0.47*	− 0.37	0.10	0.29	− 0.50*
	IIX	− 0.36	− 0.05	0.55*	0.05	0.33	0.61**	− 0.54*	− 0.58*	− 0.11	0.27
	IIXB	0.42	0.06	− 0.27	0.41	0.10	− 0.42	0.07	0.68**	0.28	− 0.27
	IIB	0.20	− 0.06	− 0.58*	− 0.47*	− 0.50*	− 0.36	0.71**	0.35	− 0.08	− 0.20

^1)^Warner–Bratzler shear force.

*P < 0.05; **P < 0.01.

## Discussion

Fresh meat quality varies among pork muscles owing to differences in their muscle fiber characteristics, which are determined by the physiological functions^8,12,21^. Our results on muscle fiber characteristics and their relationship with fresh meat quality among four different muscles are consistent with previous studies, regardless of animal species^22–25^. PM had a higher composition of oxidative fibers I or IIA, compared to that observed in SM or ST, resulting in a redder meat with better water-holding capacity and toughness. Despite its low composition of oxidative fibers, LTL showed similar values of toughness and water-holding capacity than those found in to PM. Some studies suggest that this difference may be due to a higher content of IMF in LTL than that found in PM^26,27^.

We found quality differences among the four muscles after freezing/thawing. In other words, all quality characteristics, except for pH, were affected by freezing/thawing. The pH of meat tends to decrease after freezing/thawing as hydrogen ions are released from the thawed meat, and thus increasing the solute concentration by eliminating the exudate from the meat^15^. However, the present study did not show any effect of freezing/thawing on the pH of meat, and this unique tendency seems to be attributable to the different meat species (pork) from that in the previous study (ostrich). In addition, the trend of quality changes caused by freezing/thawing was not the same among four muscles, due to their different freezing-susceptibility. As observed in the interaction between freezing/thawing and muscle type, meat color, toughness, and purge loss were altered differently by freezing/thawing among the different types of muscle. Since the composition of PM was lower in fiber IIB and higher in I and IIA compared to the composition of all other muscles, PM showed a lower freezing-susceptibility. As demonstrated previously, fiber type I is more stable to freezing/thawing than fiber types IIA or IIX, due to the differences in the size of structural proteins and Z-disk width^22,28^. Although both ST and PM had a similar composition in relative number and area of muscle fiber, ST showed differences in tenderness (increase in WBSF) and water-holding capacity (increase in purge loss) after freezing/thawing. Remarkably, we found that the size of muscle fibers in ST was larger than the size observed in PM, regardless of muscle fiber types. These findings suggested that muscle fiber size and muscle fiber composition are related to freezing-susceptibility, as observed the correlations between MFCs and the differences in meat quality between fresh and frozen-thawed pork. The muscle fiber composition of type IIA is closely related to purge loss and meat color traits, especially redness, yellowness, and chroma values. In addition, the correlations between MFC of type I and IIA (negative correlations) and purge loss and between MFC of type IIB and meat color (redness, yellowness, and chroma) and purge loss support the different susceptibility to freezing of PM to the other muscles.

Freezing and thawing reduce the meat water-holding capacity due to the formation of ice crystals, which damage the muscle fiber structure^15,29–31^. In addition, the modification and denaturation of meat proteins are accompanied by a reduction of water-holding capacity^32^. In this study, the purge loss due to freezing/thawing increased in all muscles. Therefore, a high amount (over 6.0%) of meat exudates was released from SM or ST. Consequently, SM and ST turned tougher after freezing/thawing. It has been demonstrated that a larger size or a higher composition of fiber IIB increases purge loss, cooking loss, and shear force^5,17,25,33,34^. In addition, pork meat exudates during cold storage and additional exudates are released during freezing/thawing, increasing the WBSF values^35^. These findings are consistent with our observations in SM and ST, which have large IIB fibers. Due to the breakage of type II fibers by freezing, it could be expected higher toughness values in SM or ST after freezing/thawing. However, their toughness increased due to exudation. These results are supported by the correlation between purge loss and the MFC of type I, IIA, and IIB. Moreover, purge loss was negatively correlated with type I and IIA, but positively correlated with type IIB. These correlations are consistent with the observed large size of IIB fibers in LTL, SM, and ST; higher composition of IIB fibers in LTL and SM; and lower composition of type IIA fibers in LTL, SM, and ST than in PM.

Despite LTL and SM showing a similar muscle fiber composition, we found different trends in their quality changes caused by freezing/thawing. In other words, all color traits of LTL were affected by freezing/thawing, whereas SM color was more stable compared with LTL color traits. Moreover, freezing/thawing considerably affected both water-holding capacity (especially purge loss) and WBSF in SM. The main difference between LTL and SM was the size of all muscle fiber types except for type IIB. According to the correlation between MFC and meat quality traits, fiber size of type IIX, IIXB, and IIB showed a relationship with redness or yellowness, while fiber compositions of I, IIA, IIX, and IIB were correlated with purge loss. These correlations support the more stable color in SM than in LTL and susceptibility to water-holding capacity in SM.

On the other hand, LTL and ST showed a similar fiber sizes but different muscle fiber compositions. LTL and ST showed similar color changes but different changes in WBSF. Quality changes in LTL, which are distinguishable from those in SM and ST, indicate that muscle fiber characteristics are comprehensively associated with pork quality changes due to freezing/thawing. Besides muscle fiber characteristics, other meat components such as moisture, IMF, and connective tissues are also responsible for meat tenderness and their composition can affect the stability of meat quality during freezing^26,36–38^.

## Materials and methods

### Sample preparation

Pork loin (M. *longissimus thoascis et lumborum*, LTL), tenderloin (M. *psoas major*, PM), and ham (inside, M. *semimembranosus*, SM and eye, M. *semitendinosus*, ST) were obtained from the left side of the carcasses (n = 20, castrated crossbred, Landrace × Yorkshire × Duroc, 78.5 ± 2.9 kg carcass weight) at 24 h postmortem at a commercial slaughter house. All samples were prepared as whole muscles with excessive fat and connective tissues trimmed off. Prior to packaging, samples (1 × 1 × 0.5 cm) for immunohistochemistry were taken from each muscle after removing approximately 2.5 cm from the end of the muscle. Each muscle sample was taken from the same direction for each muscle type, immediately frozen in 2-methylbutane chilled with liquid nitrogen, and stored at − 78 °C for further analysis. For each cut, muscles were individually weighed, vacuum-packed (1.0 bar; HFV-600, Hankook Fujee Industries Co., Ltd., Hwaseong, Korea), and randomly allocated to two groups (10 muscles per group): 1) fresh, cold storage at 4 °C for 7 days, and 2) frozen/thawed, frozen and stored at − 20 °C for 5 days and then thawed at 4 °C for 2 days. After seven days of storage, all muscles were removed from the packages for analyses of meat quality.

### Proximate composition

Moisture, crude protein, and crude ash were measured using the AOAC^39^ method. For intramuscular fat measurement, the method by Folch et al.^40^. with modifications was used. In brief, five grams of each sample were homogenized in 25 mL of Folch I solution (chloroform: methanol = 2:1, v/v) at 8,000 rpm (T18, IKA Works GmbH & Co., Staufen, Germany) for 30 s and stirred for 5 h at room temperature. The homogenates were filtered through Whatman No. 1 filter paper and allowed to separate into two layers for 2 h at room temperature after adding 6 mL of 0.88% NaCl. The upper layer was removed, and the lower layer was transferred to a glass bottle. After solvent removal using nitrogen gas, the remaining crude fat in the glass bottle was weighed and the value was expressed as a percentage of the initial sample weight (5 g).

### pH and meat color

Three grams of each sample were homogenized with 27 mL of deionized water and the pH was measured using a pH meter (MP230, Mettler-Toledo, Greifensee, Switzerland). To measure meat color, each muscle was cut in the center and the cut surface was exposed to air for 30 min to oxygenate myoglobin. A colorimeter (CR-400, Minolta Co., Tokyo, Japan) was calibrated with a white ceramic plate (Y = 93.5, x = 0.3132, y = 0.3198) and used to determine the color of the cut surface. A Commission Internationale de l'Eclairage (CIE, 1978) system was used to evaluate lightness (CIE L*), redness (CIE a*), yellowness (CIE b*), chroma, and hue angle.

### Water-holding capacity

To determine water-holding capacity, each sample was analyzed for purge loss, drip loss, and cooking loss. Each muscle was weighed after removing from the package, and the loss of exudates during storage was expressed as a percentage of the initial weight of the muscle. To measure drip loss, approximately 50 g of each muscle was suspended in a plastic bag for 24 h at 4 °C according to the procedure of Honikel^41^ with modifications. Each piece was weighed and the drip loss was expressed as a percentage of the initial weight of the meat piece. To measure cooking loss, approximately 50 g of each muscle was packed in a plastic bag and cooked in a water bath at 75 °C. When the internal temperature of the muscle piece reached 70 °C, it was removed from the water bath, cooled to room temperature (25 °C) for approximately 30 min, and weighed. The cooking loss was expressed as a percentage of the initial weight of the piece prior to cooking.

### Toughness

To determine meat toughness, three cores (1.3 cm in diameter) were obtained from each cooked sample. The cores were removed parallel to the orientation of the muscle fiber and cut vertically using an Instron (Model 4400, Instron Corp., MA, USA) with a Warner–Bratzler shear blade. Toughness was expressed as the average Warner–Bratzler shear force (WBSF, N/cm^2^) of the three cores for each cooked sample.

### Immunohistochemistry

For the analysis of muscle fiber characteristics (MFC), immunohistochemistry was conducted using four anti-myosin heavy chain (MHC) antibodies specific to one or more MHCs: BA-F8, anti-MHC slow; SC-71, anti-MHC 2a and 2x; BF-35, anti-MHC slow and 2a; BF-F3, anti-MHC 2b. All anti-MHCs were applied to immunohistochemistry with 5.0 µg/mL of concentration according to the manufacturer’s instructions (DSHB (Iowa City, IA, USA). Four different types of secondary antibodies (AlexaFluor647 IgG_2b_, AlexaFluor594 IgG_1_, AlexaFluor488 IgG_1_, and AlexaFluor405 IgM; Thermo Fisher Scientific, Waltham, MA, USA) were used to visualize muscle fibers. Immunostaining and muscle fiber typing were conducted in accordance with the procedure of Song et al.^22^. with modifications. In brief, transverse Sects. (10 µm in thickness) were prepared using a cryostat microtome (CM 1860, Leica Biosystems, Nussloch, Germany) at − 20 °C. After drying at room temperature for 10 min, sections were blocked in 10% normal goat serum (Cell Signaling Technology, Danvers, MA, USA) for 1 h at room temperature. All sections were incubated with primary and secondary antibodies for 1 h each at room temperature in a dark container, then washed in phosphate-buffered saline three times for 5 min each. Images from three different regions of each section were captured using a confocal scanning laser microscope (TCS SP8 STED, Leica Biosystems, Wetzlar, Germany).

### Muscle fiber characteristics (MFC)

Images were analyzed using Image-Pro Plus 5.1 (Media Cybernetic Inc., Rockville, MD, USA). According to the distribution of MHCs^24^, muscle fibers were classified into six types including four pure (I, IIA, IIX, and IIB) and two hybrid (IIAX and IIXB) types: I, specified by BA-F8 and BF-35; IIA, specified by SC-71 and BF-35; IIX, specified by SC-71; IIB, specified by BF-F3; IIAX, specified by SC-71 (weak intensity) and BF-35; IIXB, specified by SC-71 (weak intensity) and BF-F3. Approximately 600 fibers for each section were counted, and the numbers of different muscle fibers and the cross-sectional area (CSA, µm^2^) were measured. The relative number and area for each fiber type were presented as proportions (%) to the total number and area of muscle fibers, respectively.

### Statistical analysis

Data for proximate composition, pH, meat color, drip loss, cooking loss, and WBSF were collected from experimental triplicates for each muscle. Results for the MFC were collected from three different regions of each section. All data were presented as means and standard errors obtained from 10 muscles for each group (fresh and frozen/thawed) per muscle type (LTL, PM, SM, and ST). Statistical analysis was conducted using SAS 9.4 software (SAS Institute, Cary, NC, USA). The proximate composition between the four types of muscles was compared using the GLM procedure. Two-way ANOVA was performed to test the effects of muscle type, freezing/thawing, and their combination on pork quality. The relative size of muscle fiber was compared between muscle fiber types (I, IIA, IIAX, IIX, IIXB, and IIB) within the same muscle, and CSA, relative number and area were compared between muscle types within the same type of muscle fiber using the GLM procedure. The CORR procedure was used to evaluate the relationship between MFC and differences in meat quality characteristics between fresh and frozen-thawed pork. Significance was considered at *P* < 0.05, *P* < 0.01, and *P* < 0.0001.

## Conclusions

Among four different pork cuts consisting of a single muscle, PM was the most stable after freezing/thawing, whereas the other cuts showed different trends in meat quality changes due to their different freezing-susceptibility. Such differences are associated with muscle fiber size and compositions, especially the size of fiber IIB or composition of glycolytic fiber types, since they are related to deterioration of water-holding capacity and toughness during freezing/thawing, whereas a low composition of oxidative fibers is associated with discoloration caused by freezing/thawing. Although muscle fiber characteristics are partially similar among meat cuts, it is recommended to evaluate the muscle fiber characteristics to ensure the stability of pork quality during freezing/thawing. We conclude that the deterioration of meat quality observed during frozen storage can be prevented by considering muscle fiber characteristics of each pork cut.
